# Content-Specific Interpretation Bias in Children with Varying Levels of Anxiety: The Role of Gender and Age

**DOI:** 10.1007/s10578-019-00883-8

**Published:** 2019-03-16

**Authors:** Lynn Mobach, Mike Rinck, Eni S. Becker, Jennifer L. Hudson, Anke M. Klein

**Affiliations:** 1 Department of Psychology, Behavioural Science Institute, Radboud University, Montessorilaan, 6525 HR Nijmegen, The Netherlands; 2 Department of Psychology, Centre for Emotional Health, Macquarie University, Sydney, Australia; 3 Developmental Psychology, University of Amsterdam, Amsterdam, The Netherlands

**Keywords:** Childhood anxiety, Interpretation bias, Content-specificity, Gender, Age

## Abstract

The current study examined whether children varying in their levels of social anxiety, separation anxiety and spider fear exhibit a negative interpretation bias specific for their fears. Furthermore, age and gender were assessed as moderators of this relation. Children (*N* = 603) of the age of 7–12 years were asked to solve ambiguous scenarios reflecting social threat, separation threat or spider threat. Children’s levels of anxiety were assessed with self-report questionnaires. Results indicated that children scoring higher on self-reported social anxiety, separation anxiety or spider fear, displayed a negative interpretation bias for the threat-scenarios pertaining to their specific anxiety or fear, even after controlling for comorbidity with other anxiety subtypes. Contrary to our hypotheses, we did not find moderating effects of age or gender. These results indicate that even in a community sample, content-specificity of negative interpretation biases is present.

## Introduction

The role of biased cognitive processing in the onset and maintenance of anxiety has been emphasized in many cognitive models [1–4]. These biased cognitive processes are theorized to be the result of overactive schemas that are organised around the theme of threat. These schemas make anxious children more sensitive to the perception of threat [3]. One of the biased cognitive processes that has a central role in cognitive behavioural models of anxiety is a negative interpretation bias [1, 4]. This bias is the tendency to interpret ambiguous situations as negative and/or threatening.

It is reasonably well-established that anxiety is related to interpretation bias in adults [5, 7], adolescents and school-aged children [6, 8, 9]. To illustrate, a recent meta-analysis looked into the relation between anxiety and negative interpretation biases in children and adolescents under the age of 22 [8]. Stuijfzand et al. [8] included a total of 77 studies of which 18 studies included clinical samples and 57 studies included community samples. The authors found a medium-sized effect (*d* = 0.62) for the relation between anxiety and interpretation bias, robust for both clinical and community samples.

Interpretation bias is assumed to be explained by specific anxiety states (i.e., content-specificity hypothesis) [10], rather than by a general vulnerability to threat. This hypothesis suggests that the relation between anxiety and interpretation bias is stronger for ambiguous situations that match specific anxiety types. For example, a child with a social anxiety disorder would display a stronger bias for ambiguous social situations than for ambiguous situations involving spiders. A general threat bias indicates that anxious children would display a threat bias for all ambiguous situations, not dependent on their specific anxiety state. Theoretically, content-specificity of a negative interpretation bias makes sense as this would indicate that threat schemas of a child with social anxiety disorder would surround the theme of social threat and would make this child more sensitive to perceive threat in ambiguous social situations [3, 10]. This hypothesis received support from the meta-analysis by Stuijfzand et al. [8]: the relation between anxiety and interpretation bias increased in strength when the specific anxiety subtype was matched with the scenario content [7, 8].

However, the content-specificity hypothesis has been investigated only in very few studies with child samples. Where children have been the focus of studies assessing the relation between anxiety and content-specific interpretation bias, most research has looked into social anxiety or general anxiety [8]. Stuijfzand et al. [8] noted that their findings were mostly driven by the relation between social anxiety and social scenarios compared to non-social scenarios. There were not enough studies providing effect sizes on anxiety subtypes other than social anxiety or separation anxiety. The authors therefore concluded that it is too early to generalize these findings to all anxiety disorders in this age group. Studying whether a negative interpretation bias is specific to the anxiety subtype in children is crucial in establishing whether treatment should target interpretations specific to the anxiety diagnosis of the child. Currently, children with anxiety disorders all receive the same transdiagnostic cognitive behavioural treatment program [11], which means that treatment is not focused on cognitive content specific to the anxiety disorder. Instead, treatment focuses on general information processing biases, such as a general interpretation bias towards threat. Although cognitive behavioural therapy is relatively effective, there is still much room for improvement [11]. Importantly, clinical trials for adult and adolescent populations have demonstrated that disorder specific programs, focused on specific cognitive content of the anxiety disorder, resulted in significantly better treatment outcomes [12, 13]. In line with cognitive theories, these findings suggest that the specific cognitive content of the anxiety disorder affects the maintenance of anxiety symptoms and that targeting this content might stop the chronic overactivation of threat schemas [1–4]. However, more research is needed on multiple anxiety subtypes in children [8] to generalize the findings for this age group and to be able to define the focus of treatment. Therefore, the goal of the current study was to test the content-specificity hypothesis in other fears and anxieties in a community sample of children.

Although research on other childhood anxiety subtypes is relatively scarce, there are a few studies that focused on the content-specificity of interpretation bias for other prevalent childhood anxiety subtypes. These included separation anxiety and spider fear, finding mixed results [14–19]. For example, Bögels et al. [14] found content-specific interpretation biases in children (aged 7–12) with subclinical levels of social anxiety and separation anxiety, but not for generalized anxiety disorder (GAD) using an ambiguous scenario test. An explanation for the latter finding could be that GAD is characterised by excessive worrying about a wide array of topics, such as the child’s health, homework, and a general sense of feeling safe. As these topics can vary extensively between individuals diagnosed with the same disorder, it might have been harder to create stories covering worry content that is specific for GAD [20]. Muris and colleagues [21] found that the general level of anxiety better accounted for the number of threat perceptions in children (aged 8–13), compared to specific anxiety symptoms, finding evidence for a general threat bias instead of for content-specificity of a negative interpretation bias. Muris et al. [21] also used a story-based task, but one explanation for the lack of specificity that the authors discussed is that some of their stories might have triggered threat perception in children high on both social and separation anxiety symptoms.

Furthermore, because comorbidity between social, separation and generalized anxiety symptoms is generally high [22], another explanation for the differences in findings could be that co-morbidity might have played a role. For example, Klein et al. [18] studied social anxiety, generalized anxiety and separation anxiety in clinically anxious children using a story-based task, finding that there was some overlap in the correlations between the different anxieties and the non-congruent subscales of the story task. However, when they conducted regression analyses where they controlled for co-morbidity, only the anxiety matching the specific subscale of the stories task was a significant predictor. This indicates that different anxieties do show overlap, but also that interpretation biases are content-specific. Clearly more research is needed to test the content-specificity hypothesis in other childhood fears and anxieties other than social anxiety while also taking co-morbidity into account. The current study therefore focused on children with varying levels of social anxiety, separation anxiety and spider fear, as these represent three of the most prevalent anxieties in childhood, while also controlling for co-morbid symptoms in the analyses [23, 24].

Besides the need to replicate results and elucidate whether content-specificity is present for multiple, highly prevalent anxiety subtypes in childhood, an important extension is to identify variables that moderate whether content-specificity of interpretation bias is present. One potentially important developmental factor that might moderate the relation between anxiety subtypes and the content-specificity of interpretation biases, is age. Indeed, Stuijfzand et al. [8] found in their meta-analysis that the relation between anxiety and interpretation bias increased in strength as children were older, indicating that taking a developmental approach in assessing this relation is needed to establish whether targeting negative interpretation biases for specific anxiety subtypes in treatment, might be more appropriate for a specific age group.

Unfortunately, most studies of content-specificity in different anxiety subtypes have not assessed age as a moderator [16, 18, 19, 25]. As normative changes in cognitions tend to take place in childhood and adolescence, this is surprising. The studies that did assess age as a moderator included a broad age range, but were underpowered to assess specific developmental patterns for each anxiety subtype [8, 26]. Waite et al. [26] compared threat interpretations between children (aged 7–10) and adolescents (aged 13–16) with and without anxiety disorders. They found that anxious adolescents showed more threat interpretations than non-anxious adolescents, but that no such difference existed between the non-anxious and anxious younger groups. Furthermore, there was no difference between the children and the anxious adolescents in the number of threat interpretations they made. This indicates that, even for anxious younger children, the number of threat interpretations may not be different from anxious adolescents or non-anxious children. Waite et al. [26] speculated about a possible explanation: When reaching adolescence, non-anxious children may be better able to inhibit the negative interpretations as a result of stabilizing thinking styles and a larger body of experiences to derive interpretations from.

However, as also suggested by Stuijfzand et al. [8], another possibility is that age interacts with specific anxiety symptom types over the course of development. Waite et al. [26] did not assess this possibility because of a lack of power. So far, the studies discussed above that have looked at the role of age in relation to interpretation bias consist of a meta-analysis that has investigated the role of age across studies, but there has only been one empirical study so far investigating the role of age. This study, however, excluded ages 10–12 and has compared two distinct age groups [26]. Up to now, there have not been any individual empirical studies looking at the possible moderating role of age in children aged 7–12. Importantly, research does indicate that children in the age of 7–12 years are in a critical developmental period when it comes to cognitive functions and as a result, this may influence the development of information processing biases [27–29].

A review into the role of development in relation to information processing biases to threat in children [27] suggests that biases already occur in early childhood, but that these seem to change as a function of the child’s increasing age. Research on attention bias to threat indeed suggests that the critical developmental period arises in middle childhood (ages 7–12) [29]. Kindt and van den Hout [29] formulated the inhibition hypothesis, which suggests that middle childhood is crucial for children to learn to inhibit automatic threat processing. They postulate that children who do not learn to inhibit automatic threat processing, have a larger chance of developing more anxiety symptoms later on. This is in line with research suggesting that information processing capacity and executive functioning processes, such as inhibition and task switching is rather limited in younger children, but that these cognitive functions show rapid improvements from 7 to 11 years [30]. As these cognitive functions are paramount to be able to regulate emotional responses and cognitions associated with emotional responses, it is very likely that this age group will already show differentiation of interpretation bias to threat from 7 to 12 years old [31, 32]. Unfortunately, the review also indicates that this conclusion is mostly based on research of attention bias, whereas research into interpretation bias, especially in the age group of 7–12, is still scarce [27]. Therefore, it is important to investigate the possible moderating role of age for interpretation bias to threat in the ages of 7–12 years as well. The current study assessed age as a moderator of the relation between specific anxiety symptom types (social anxiety, separation anxiety and spider fear) and content-specificity of interpretation bias in children in middle childhood.

Furthermore, it is well-known that across all ages, most anxiety subtypes are more frequent in girls [33, 34]. Previous research has found that girls chose more negative interpretations in social situations, but that there were no differences between boys and girls in non-social situations [35, 36]. Importantly, research has found that gender predicts outcome after cognitive behavioural therapy for children with anxiety disorders, such that girls tended to have poorer outcomes than boys [37]. However, another study was not able to replicate this finding [38]. Results seem to be mixed and it is not clear why gender might be a predictor of treatment outcome. Therefore, it is important to further examine whether gender is a moderator in important maintaining mechanisms of anxiety, such as a negative interpretation bias. Surprisingly, however, gender has not been assessed as a moderator of the relation between interpretation bias specificity and anxiety subtypes [8]. Therefore, the current study also assessed gender as a moderator of this relation, to establish whether targeting negative interpretation biases related to specific anxiety subtypes in treatment may be more appropriate for girls or boys.

The present study had three principle aims. The first aim was to replicate the finding found in the meta-analysis by Stuijfzand et al. [8] that interpretation bias is content-specific for social anxiety symptoms. Second, we tested whether interpretation bias is also content-specific for separation anxiety and spider fear [16, 18, 19]. The third aim was to assess age and gender as moderators of the relation between interpretation bias and anxiety. It was hypothesized that interpretation bias is content-specific for social anxiety [8], as well as for separation anxiety and spider fear [16, 18, 19]. Furthermore, it was hypothesized that both age and gender will moderate the relation between interpretation bias and anxiety. Specifically, with regards to age, we expected that the strength of the relation would increase with age [8, 39]. With regard to gender, we expected that the relation would be stronger for girls than for boys [35, 36].

## Methods

### Participants

Children between the ages of 7 and 12 years were recruited from 15 regular elementary schools throughout the Netherlands. As the current study was part of a larger project focused at assessing emotion processing, social processes and childhood anxiety, the children also completed other measures (a full list of all the measures can be obtained from the authors). Two other published papers about different topics were based on the same participant group [40, 41]. The current study was conducted in 11 of the 15 schools among 605 children. The Ethics Committee of the Faculty of Social Sciences of Radboud University Nijmegen, the Netherlands, approved this study (ECSW2016-2811-447R3001).

After the school directors approved data collection at the schools, active consent was obtained from the parents. Before the start of the session, children could indicate if they wanted to participate, and children aged 12 or older were also asked to give active consent. As there were only two 7-year-old children, we excluded these children from the analyses in which we assessed age as a moderator. However, we included these children for the correlations. The final sample consisted of a total of 603 children (312 girls) aged 7–12 (*M* = 9.79, *SD* = 1.10). Of these, 582 were born in the Netherlands and 20 were born outside of the Netherlands. One child did not provide an answer to this question.

### Materials

#### Interpretation Task

The interpretation task consisted of 15 multiple-choice scenarios, with 5 each related to social threat, separation threat, and spider threat (see Table 1 for sample scenarios of each category). Each scenario contained 4 short sentences and was ambiguous in nature. Children could choose from 4 possible interpretations: a positive, neutral, mildly negative or strongly negative interpretation (see Table 1 for sample interpretations). The 15 scenarios were selected and adapted from existing scenarios [14, 16, 18, 19, 21, 42–44]. Children were instructed to imagine themselves as being the main character in the situation described in the scenario. Scenarios were presented in a fixed order such that the first story was a social threat scenario, the second was a separation threat scenario, the third was a spider threat scenario and the fourth was a social threat scenario again. The endings were randomized per category and per story to ensure that the positive, neutral, neutral/negative and negative endings were presented equally often as the first, second, third, and fourth option, respectively. Interpretation bias was operationalized as the number of times the children chose the most negative interpretation within the category. This resulted in three interpretation bias scores: a social threat score (ω = 0.69, CI [0.65, 0.72]) [45, 46], a separation threat score (ω = 0.67, CI [0.63, 0.71]) and a spider threat score (ω = 0.73, CI [0.70, 0.77]) with values per threat score ranging between 0 and 3.

**Table 1:** Sample scenarios from the interpretation task: a social threat, separation threat, and spider threat scenario with the endings

Scenario	Endings
Social threat “Presentation” Today I have to give a presentation about my hobby at school. All the kids are sitting in their seats. When I am in front of the class, I think that …	Positive I am excited to tell my classmates about my hobby Neutral I will also show some pictures Moderately negative I will forget what I wanted to say Strongly negative Everyone thinks I suck
Separation threat “The movies” Mom and dad will go to the movies tonight. They will be home very late. My grandmother will watch me. I think that …	Positive it will be a fun night with grandma Neutral it is an interesting movie Mildly negative I cannot sleep without mom and dad Strongly negative something bad will happen to mom and dad
Spider threat “Cleaning my room” Mom wants me to clean my room. Whilst I am cleaning, I see something dusty beneath my desk. When I try to grab it, something tickles my hand. I think that …	Positive it is the fur from my favourite stuffed animal and that I am happy I found it Neutral it is dust Mildly negative it is a spider web and the spider might be around somewhere Strongly negative it is a big spider who bites me

#### Social Anxiety Scale for Children-Revised (SASC-R) [47]

The SASC-R is a self-report questionnaire consisting of 18 items which measure social anxiety symptoms in children. The items are scored on a 5-point Likert scale ranging from 0 (*not at all*) to 4 (*always*). The SASC-R includes three domains pertaining to social anxiety: Fear of negative evaluation, social avoidance and distress in new situations, and general social avoidance and distress. Total scores were obtained by summing up the scores on the items. Higher scores indicate more social anxiety. Construct validity and internal consistency (ranging from α = 0.69 to 0.86) have been previously reported as acceptable [47]. Internal consistency for the entire scale in the current study was good (ω = 0.91, CI [0.90, 0.92]).

#### Spence Children’s Anxiety Scale, Child version, Separation Anxiety Subscale (SCAS-C) [48]

The Dutch version of the separation anxiety subscale of the SCAS-C was used to assess separation anxiety in children. The separation anxiety subscale is a self-report measure and consists of 6 items which are scored on a 4-point Likert scale ranging from 0 (*never*) to 3 (*always*). A total score was obtained by summing up the scores on all items. Higher scores indicate more separation anxiety symptoms. Construct validity, internal consistency (*α* = 0.70) and test–retest reliability (*r* = 0.57) have been previously reported as acceptable [48]. Internal consistency in the current study was also acceptable (ω = 0.72, CI [0.68, 0.75]).

#### Spider Anxiety and Disgust Screening for Children (SADS-C) [49]

The SADS-C is a four-item self-report questionnaire which assesses the extent to which children experience fear, physical arousal, avoidance and disgust regarding spiders. Children report on a 5-point Likert scale ranging from 0 (*not at all true for me*) to 4 (*very true for me*). Scores on the items are summed up to create a total score. Higher scores indicate more fear for spiders. Construct validity, internal consistency (*α* = 0.88) and test–retest reliability (*r* = 0.91) have been previously reported as satisfactory [49]. Internal consistency in the current study was good (ω = 0.91, CI [0.90, 0.92]).

### Procedure

Children participated in the tasks and questionnaires in their classroom environment. Children were seated in such a way that they could not see each other’s’ answers. The children first filled out the interpretation task followed by the questionnaires. Overall, tasks and questionnaires took approximately 50–60 min to complete. Children received a participation certificate and a small gift (worth approx. 1 Euro). The school directors received a school-wide report and were additionally offered a workshop on childhood anxiety.

### Data Analysis

First, descriptive statistics of the anxiety variables were explored separately for each age group and gender. Second, partial correlations between the anxiety variables and the threat scores were calculated to explore the relation between these variables. Correlations were controlled for age and were examined separately for boys and girls. Third, an assumption check was performed for the variables that were included in the regression analyses. Visual inspection indicated that both separation anxiety and spider fear were skewed, and they were therefore transformed to approximate normality. Separation anxiety was log-transformed, skewness = 1.31 (*SE* = 0.10), kurtosis = 2.29 (*SE* = 0.20). Spider fear was square root transformed, skewness = − 0.31 (*SE* = 0.10), kurtosis = − 1.13 (*SE* = 0.20). Social anxiety was approximately normally distributed, skewness = 0.66 (*SE* = 0.10), kurtosis = 0.14 (*SE* = 0.20). There was no evidence for multicollinearity between the predictors in any of the regression analyses (VIF < 2; *r*’s < 0.60).

Next, to test the hypothesis that interpretation bias is content-specific, three hierarchical regression analyses were conducted. The first hierarchical regression included social anxiety level as the criterion. In the first step, gender, separation anxiety level, and spider anxiety level were added to control for these variables. In the second step, interpretation bias scores for social threat, separation threat, and spider threat scenarios were added as predictors, to test if these biases would predict variance in the social anxiety level over and above the variance predicted by the predictors in the first step. The second and third hierarchical regression analyses included separation anxiety level and spider fear level as the criteria, respectively. The predictors in the first steps were equal for both regression analyses, except for the addition of the anxiety levels: The regression analysis with separation anxiety as the criterion included spider fear and social anxiety as predictors in the first step, and the regression analysis with spider fear as the criterion included social anxiety and separation anxiety as predictors in the first step. However, in the first step of the regression analysis with separation anxiety as the criterion, age was added to control for this variable as a one-way ANOVA showed significant differences between children of different ages (8/9/10/11/12) for separation anxiety symptoms (see below for statistics). The second steps were identical to the first regression analysis.

Finally, to test the hypothesis that gender and age would moderate the relation between interpretation bias and anxiety, we performed three moderation analyses in PROCESS [50]. The first moderation analysis included social anxiety as the outcome variable, the threat score for social anxiety as the predictor and gender and age as moderators. We included age in months instead of years in all the moderation analyses in order to measure age more precisely. The second moderation analysis included separation anxiety as the outcome variable, the threat score for separation anxiety as the predictor and gender and age as moderators. The third moderation analysis included spider fear as the outcome variable, the threat score for spider fear as the predictor and gender and age as moderators. All moderation analyses used bias-corrected 95% confidence intervals using bootstrapping with 5000 resamples (model 4) [50].

## Results

### Descriptives

#### Self-reported Anxiety Symptoms

Table 2 shows the means and standard deviations for self-reported social anxiety, separation anxiety and spider fear. To give an indication of the relevance of the reported anxiety symptoms, 15.5% of the children scored above the clinical cut-off for social anxiety symptoms [47], 37.4% scored above the mean score of the separation anxiety subscale [48], and 27.9% of the children scored above the mean score of the spider fear symptoms [49]. A one-way analysis of variance (ANOVA) revealed a significant difference between boys and girls on self-reported social anxiety, *F*(1,599) = 34.762, *p* < 0.001, separation anxiety, *F*(1,599) = 36.293, *p* < 0.001, and spider fear, *F*(1,597) = 123.539, *p* < 0.001. As is often found in the literature [51], girls scored higher than boys on all three anxiety variables: On social anxiety, girls (*M* = 2.21, *SD* = 0.65) scored higher than boys (*M* = 1.90, *SD* = 0.65), on separation anxiety^1^, girls (*M* = 0.64, *SD* = 0.49) scored higher than boys (*M* = 0.42, *SD* = 0.40), and girls (*M* = 2.06, *SD* = 1.28) scored higher than boys (*M* = 0.96, *SD* = 1.07) on spider fear as well. We therefore controlled for gender in all analyses. Another one-way ANOVA showed a significant difference between ages (8/9/10/11/12), but only for separation anxiety, *F*(4,599) = 7.838, *p* < 0.001. Holm-Bonferroni-corrected post-hoc tests indicated that younger children (aged 8–9) were significantly different from older children (10–12): Younger children scored higher on separation anxiety than older children (see Table 2 for the means). We therefore only controlled for age in analyses where separation anxiety was used as the dependent variable.

**Table 2:** Means and standard deviations for social anxiety, separation anxiety and spider fear, separately for age group and gender

Age	Gender	Social anxiety	Separation anxiety	Spider fear
		M (SD)	M (SD)	M (SD)
8	Boys	2.13 (0.77)	0.63 (0.66)	1.04 (1.00)
	Girls	2.28 (0.69)	0.73 (0.49)	2.03 (1.29)
9	Boys	1.98 (0.66)	0.47 (0.37)	0.86 (0.97)
	Girls	2.24 (0.63)	0.78 (0.60)	2.17 (1.19)
10	Boys	1.88 (0.65)	0.42 (0.38)	0.93 (1.20)
	Girls	2.11 (0.62)	0.56 (0.40)	1.89 (1.28)
11	Boys	1.68 (0.49)	0.35 (0.33)	1.11 (1.10)
	Girls	2.26 (0.72)	0.54 (0.37)	2.17 (1.36)
12	Boys	1.80 (0.67)	0.20 (0.21)	1.04 (0.99)
	Girls	2.25 (0.57)	0.45 (0.25)	2.02 (1.42)

#### Threat Interpretations

For the social threat scenarios, overall, most children chose the positive interpretations (*M* = 2.24, *SD* = 1.33), then the neutral interpretations (*M* = 1.29, *SD* = 0.97), then the mildly negative interpretations (*M* = 0.91, *SD* = 0.83), and finally the strongly negative interpretations (*M* = 0.54, *SD* = 0.93). For the separation threat scenarios, overall, most children again chose the positive interpretations (*M* = 2.95, *SD* = 1.00), then the mildly negative interpretations (*M* = 0.96, *SD* = 0.96), then the neutral interpretations (*M* = 0.84, *SD* = 0.77), and finally the strongly negative interpretations (*M* = 0.23, *SD* = 0.57). For the spider threat scenarios, overall, most children chose the neutral interpretations (*M* = 2.09, *SD* = 1.37), then, the mildly negative interpretations (*M* = 1.44, *SD* = 1.44), then the positive interpretations (*M* = 1.17, *SD* = 1.12), and finally the strongly negative interpretations (*M* = 0.27, *SD* = 0.71).

Partial correlations between the anxiety variables and the threat scores, separately for boys and girls and controlled for age, are displayed in Table 3. In general, all three biases scores were significantly and positively correlated with all corresponding and non-corresponding anxiety variables.

**Table 3:** Partial correlations for the anxiety scores and the threat scores separately for boys and girls, controlled for age

	1.	2.	3.	4.	5.
Boys					
1. Social anxiety					
2. Separation anxiety	0.56***				
3. Spider fear	0.34***	0.31***			
4. SA-threat score	0.40***	0.22***	0.16**		
5. SEP-threat score	0.30***	0.43***	0.21***	0.42***	
6. SPID-threat score	0.33***	0.20**	0.46***	0.37***	0.37***
Girls					
1. Social anxiety					
2. Separation anxiety	0.47***				
3. Spider fear	0.22***	0.28***			
4. SA-threat score	0.50***	0.25***	0.11*		
5. SEP-threat score	0.31***	0.45***	0.16**	0.36***	
6. SPID-threat score	0.21***	0.18***	0.53***	0.34***	0.16**

*SA* social anxiety, *SEP* separation anxiety, *SPID* spider fear

*p < 0.05; **p < 0.01; ***p < 0.001

### Regression Analyses

In order to test the content-specificity hypothesis for the three anxiety subtypes, three two-step hierarchical regression analyses were conducted. For social anxiety, the first model including gender, separation anxiety and spider fear, explained 31% of the total variance in social anxiety, *F*(3,594) = 88.746, *p* < 0.001. Introducing the threat scores into the second model explained an additional 8% (*F*(6,591) = 62.372, *p* < 0.001), and this change in *R*^*2*^ was significant (*p* < 0.001). However, only the social threat score made a significant contribution to the model, *β* = 0.30, *p* < 0.001 (see Table 4 for all coefficients). Thus, only the social threat interpretation bias, but not separation anxiety bias or spider fear bias, made a significant contribution to predicting social anxiety symptoms, over and above other anxiety symptoms and gender.

**Table 4:** Hierarchical regression analyses predicting social anxiety, separation anxiety, and spider fear

Step	Variables	Social anxiety					Separation anxiety					Spider fear				
		*B*	*SE*	*β*	*t*	*p*	*B*	*SE*	*β*	*t*	*p*	*B*	*SE*	*β*	*t*	*p*
1	Gender	0.094	0.051	0.070	1.85	0.065	0.004	0.003	0.045	1.22	0.224	0.451	0.049	0.341	9.19	< 0.001
	Age						− 0.001	0.000	− 0.173	− 5.18	< 0.001					
	SOC						0.028	0.002	0.442	12.45	< 0.001	0.133	0.042	0.135	3.17	0.002
	SEP	0.684	0.053	0.470	12.81	0.001						0.256	0.061	0.178	4.19	< 0.001
	SPID	0.068	0.020	0.131	3.34	0.001	0.006	0.001	0.197	5.15	< 0.001					
2	Gender	0.069	0.048	0.051	1.42	0.155	0.005	0.003	0.055	1.50	0.135	0.444	0.048	0.336	9.19	< 0.001
	Age						− 0.001	0.000	− 0.167	− 0.503	< 0.001					
	SOC						0.027	0.002	0.418	10.89	< 0.001	0.124	0.044	0.125	2.82	0.005
	SEP	0.589	0.052	0.404	11.26	< 0.001						0.214	0.061	0.149	3.50	< 0.001
	SPID	0.060	0.020	0.116	3.03	0.003	0.006	0.001	0.170	4.33	< 0.001					
	SOC-threat	0.213	0.026	0.297	8.36	< 0.001	0.000	0.002	0.003	0.078	0.938	− 0.020	0.029	− 0.029	− 0.70	0.482
	SEP-threat	− 0.006	0.041	− 0.005	− 0.15	0.883	0.007	0.003	0.092	2.59	0.010	0.006	0.044	0.005	0.14	0.888
	SPID-threat	− 0.018	0.034	− 0.019	− 0.53	0.596	0.003	0.002	0.057	1.568	0.118	0.175	0.036	0.187	4.93	< 0.001

*SOC* social anxiety, *SEP* separation anxiety, *SPID* spider fear

For separation anxiety, the first model including gender, age, social anxiety and spider fear, explained 35% of the total variance, *F*(4,586) = 80.59, *p* < 0.001. Introducing the threat scores into the second model explained an additional 2% (*F*(7,583) = 48.586, *p* < 0.001), and this change in *R*^*2*^ was significant, *p* = 0.006. However, only the separation threat score made a significant contribution (*β* = 0.09, *p* = 0.01) in predicting separation anxiety symptoms over and above the other anxiety symptoms, gender and age, whereas the threat scores for social anxiety and spider fear did not.

For spider fear, the first model including gender, social anxiety and separation anxiety, explained 24% of the variance, *F*(3,594) = 63.185, *p* < 0.001. Introducing the threat scores into the second model explained an additional 3% of the variance (*F*(6,591) = 37.099, *p* < 0.001), and this change in *R*^*2*^ was significant, *p* < 0.001. Again, only the spider threat score made a significant contribution to the model, *β* = 0.19, *p* < 0.001. It predicted spider fear over and above other anxiety symptoms and gender, whereas the threat scores for social anxiety and separation anxiety did not.

### Moderation Analyses

As the specific threat scores were the only significant predictors in the final models of the regression analyses, the three moderation models were tested with only the specific threat scores as independent variables pertaining to the specific anxiety variables as the dependent variables. We conducted three separate moderation analyses with social anxiety, separation anxiety, and spider fear as the outcome variable, respectively. In each of the moderation analyses, the threat score pertaining to that specific anxiety subtype was included as the independent variable, and gender and age were added as moderators. For the social anxiety model, we did not find a significant moderating effect of either age (*B* = 0.00, *p* = 0.948) or gender (*B* = − 0.01, *p* = *0*.862). Similarly, for the separation anxiety model, we did not find a significant moderating effect of age (*B* = − 0.00, *p* = 0.176) or gender (*B* = 0.00, *p* = 0.913). Finally, for the spider fear model, we did not find significant moderating effects of age (*B* = 0.00, *p* = 0.122) or gender (*B* = − 0.03, *p* = 0.630) either. Thus, for all anxiety subtypes, the predictive power of the corresponding content-specific anxiety threat score did not depend on gender or age.

## Discussion

The main aim of this study was to assess whether there are content-specific negative interpretation biases in children varying in social anxiety, separation anxiety and spider fear. Another aim was to assess whether gender and age are moderators of this relation. As expected, we found that children with higher scores on social anxiety, separation anxiety or spider fear displayed a negative interpretation bias only for the threat-scenarios pertaining to their specific anxiety or fear, even after controlling for comorbidity with other anxiety subtypes. Contrary to our hypotheses, neither age nor gender moderated the relation between the specific threat scores and the types of anxieties.

The results of the current study add to the growing body of evidence suggesting that anxious children have a negative interpretation bias specifically for threat-related scenarios that reflect the content of their anxiety or fear [8, 10, 14, 52]. The results also confirm the same findings found in adults [7]. The results replicate the consistent finding that children with higher scores on social anxiety display a negative interpretation bias specifically for social threat scenarios [8]. Furthermore, these results confirm earlier studies that have indicated content-specificity of a negative interpretation bias for separation anxiety [14, 18] and spider fear [16, 19]. These results indicate that overactive cognitive schemas are not focused on threat in general, but that these schemas seem to surround specific threat information. This in turn may make children more sensitive to perceiving threat specific to their anxiety symptoms [3, 10].

However, contrary to our expectations, our results do not suggest that age would moderate the relations between social anxiety, separation anxiety, and spider fear with their specific interpretation biases. Instead, the results suggest that the associations between content-specific negative interpretation biases and their corresponding anxieties do not change over the course of development in this age group. At first, this result seems to contradict earlier findings that older children tend to endorse more threat-biased interpretations than younger children [8, 26, 39]. However, it is difficult to compare our results to previous ones because the present study is the first to specifically address the moderating role of age with regard to the content-specificity of interpretation biases, rather than threat biases in general. Future research should aim to replicate our results to be able to draw stronger conclusions.

A possible explanation for the absence of a moderation by age could be that the current study included a more restricted age range (8–12) than previous studies which did find a moderating effect of age on the relation between interpretation bias and anxiety. For example, Waite et al. [26] included two age groups (7–10 and 13–16), and the meta-analysis by Stuijfzand et al. [8] encompassed an even broader age range (2–22 years). It could be that a developmental pattern would be revealed when adolescents above 12 years old or below 8 were included. It might even be the case that developmental patterns are only present for different developmental phases (early childhood versus middle childhood versus adolescence) and that within these developmental phases, there are no developmental patterns, as was previously suggested by Waite et al. [26]. We decided to choose children in middle childhood because most of the anxiety subtypes are surfacing in middle childhood, because this age group already shows a differential association between age and other information processing biases [23, 28] and because there are important cognitive developments in this age group [30–32]. However, the transition from middle childhood to adolescence marks an additional important change in developmental periods characterised by socio-emotional changes [53]. This change might be especially relevant for the lack of an age effect for social anxiety symptoms, as social anxiety symptoms usually tend to increase in adolescence rather than in the age group studied here [54, 55]. As this was the first empirical study with the aim to assess specific developmental patterns of content-specific interpretation biases, due to practical reasons, including a large child community sample with varying levels of anxiety was the only way to assess these developmental patterns with sufficient statistical power. However, future research should include a sample with a broader age range to explore whether developmental patterns are only present for between developmental phases.

Contrary to our expectations, our study did not yield any evidence of a moderating effect of gender on the relations between social anxiety, separation anxiety, and spider fear with their specific interpretation biases. Moreover, in contrast to previous studies, we did not find girls to endorse more threat interpretations for social threat situations than boys [35, 36]. A possible explanation for these findings is that the current study included a younger age group (8–12) compared to previous studies, which focused on adolescents (12–16) [35, 36]. Furthermore, these studies only found a difference between boys and girls in social threat scenarios and not in non-social threat scenarios. It is possible that gender differences in these social threat scenarios only start to surface in adolescence, as adolescent girls have been found to report more feelings of concern with regards to social inadequacy, when compared to boys in that age group [35, 36].

This study has some important strengths worth mentioning. The inclusion of a large sample enabled us to examine specific developmental patterns for specific anxiety subtypes, and their relations with the corresponding negative interpretation biases. Furthermore, this study was the first to examine the role of gender in the relation between specific anxiety subtypes and their corresponding negative interpretation biases. Importantly, this study included three of the most prevalent childhood anxiety subtypes and was the first to take comorbidity of other anxiety subtypes into account when examining the content-specificity of negative interpretation biases for specific anxieties and fears.

Some limitations of the current study should also be mentioned. First, as this was the first empirical study to assess specific developmental patterns of content-specific interpretation biases, we needed a large sample to be able to address moderating effects. We therefore included a community sample of children with varying levels of anxiety symptoms. Future research should proceed to assess the developmental patterns of content-specificity in a clinically anxious sample to be able to assess whether the current results can be generalized to clinically anxious children. Although research has shown that the effect size for the relation between interpretation bias and anxiety is robust across clinical and non-clinical samples [8], this replication is necessary because there is also research that has indicated that there may be differences between anxious and non-anxious young people when it comes to perceptions and sensitivity to threat [26]. In addition, it might be particularly important to compare developmental patterns of content-specificity between children in different developmental phases [26].

Second, for child anxiety levels, we relied solely on self-report. Research has found that in this age group, there is more discrepancy between parent- and child-report of anxiety symptoms [56, 57]. Therefore, we might have missed some children with high anxiety levels who did not report these symptoms themselves. Additionally, other parental variables were not included in the current study. Importantly, research has indicated that parental mental health is an important predictor of child anxiety and the associated interpretation bias [58]. Future research should examine parental mental health as an additional variable in the relation between content-specific interpretation bias and anxiety. Third, although the current results indicate content-specificity of negative interpretation biases, the additional amount of explained variance in anxiety symptoms by means of the specific threat scores was small. It might be the case that the specific threat scores explain less variance in an unselected community sample with children varying in levels of anxiety and fear, compared to clinically anxious children. Indeed, Klein et al. [18] found a larger amount of explained variance by content-specific threat scores of separation anxiety and social anxiety in a group of clinically anxious children. Furthermore, the small amount of additionally explained variance for negative interpretation bias when predicting anxiety symptoms is in line with previous research in children varying in levels of anxiety [16, 19].

Fourth, although this study included three of the most prevalent childhood anxiety disorders, the content-specificity hypothesis and the possible moderating role of age and gender should also be examined in other anxiety disorders. Due to constraints on time and resources, we were not able to assess whether other highly prevalent anxieties are related to content-specific interpretation biases. For example, GAD is prevalent in youth already [22], and it would be interesting to assess whether an anxiety disorder characterized by its multiple worry topics and general worry [20] would show a content-specific interpretation bias as well. Previous studies found mixed results for GAD [14, 17], with some studies reporting a lack of content-specificity for threat-related bias in GAD [14], and others finding some preliminary evidence for a content-specific interpretation bias in children varying in their levels of GAD [17]. Future research should therefore examine whether the evidence for content-specificity will hold for different anxiety disorders, including GAD.

Fifth, the stories in the interpretation bias task were not presented in a random order due to practical constraints on the data collection in the classroom: the stories were read aloud by a researcher, to ensure that all children finished all stories and that the slower-reading children could also finish in time. Presenting these stories in a non-randomized manner might have biased the results due to order effects [59]. However, because children could read the endings themselves, we were able to randomize the ending for the stories in the interpretation task per story and per category to minimize this potential bias.

Lastly, and related to the previous point, we were only able to control for the anxiety subtypes that were included in the current study, but not for other anxiety subtypes that might have played a role, such as GAD. Comorbidity of anxiety symptoms is high, even in sub-clinically anxious children [22], and GAD specifically is highly comorbid with the anxiety subtypes we have examined in the current study. It is possible that comorbidity has obscured larger predictive effects of threat scores on anxiety subtypes. Therefore, future research should include other anxiety subtypes to be able to control for comorbidity between anxiety symptoms.

In conclusion, the results of this study provide support for the existence of content-specific interpretation biases in social anxiety, separation anxiety and spider fear, found in a community sample with varying levels of these anxieties and fears. Specific fear- and anxiety-related interpretations are already present in children aged 8 and do not seem to change substantially over the course of development into early adolescence. Moreover, they do not seem to differ significantly between boys and girls of a community sample either. Content-specific negative interpretation biases for specific anxiety symptoms have been implicated in the maintenance of anxiety disorders. Therefore, more research is needed. This research should include other anxieties and fears such as GAD, it should address a broader age range, and the results should be replicated in clinical samples. If the current results hold up under these circumstances, this indicates that targeting negative interpretation bias is expected to be more efficacious when it is tailored to the specific anxiety disorder at hand. This in turn may lead to a reduction of anxiety symptoms, in line with cognitive models of anxiety disorders [1–4, 10], and in line with previous research on disorder-specific anxiety treatment in adolescents and adults [12, 13]. Importantly, if the current results hold, this would also indicate that targeting specific interpretation biases is expected to be efficacious for all age groups, and for boys and girls equally.

## Summary

To sum up, the current study found that children between 7 and 12 years of age who report higher levels of social anxiety, separation anxiety or spider fear displayed a negative interpretation bias only for the threat-scenarios pertaining to their specific anxiety or fear, even after controlling for comorbidity with other anxiety subtypes. Neither age nor gender moderated the relation between the specific threat scores and the types of anxieties. These results might have important implications for the treatment of childhood anxiety disorders: They suggest that treatment should focus more on the specific cognitive content of the negative interpretation bias per specific anxiety disorder. Further studies replicating the current results in a clinically anxious sample, in a broader age range (e.g., 7–18 years old) and in other anxiety subtypes, are highly recommended to generalize these findings.

## Footnotes

1 Non-transformed means and standard deviations for separation anxiety and spider fear are given here, for ease of interpretation.
